# Screening Pneumonia Patients for Mimivirus^1^

**DOI:** 10.3201/eid1403.071027

**Published:** 2008-03

**Authors:** Ryan K. Dare, Malinee Chittaganpitch, Dean D. Erdman

**Affiliations:** * Centers for Disease Control and Prevention, Atlanta, Georgia, USA; † Thailand Ministry of Public Health, Nonthaburi, Thailand

**Keywords:** mimivirus, pneumonia, real-time PCR, dispatch

## Abstract

*Acanthamoeba polyphaga mimivirus* (APM), a virus of free-living amebae, has reportedly caused human respiratory disease. Using 2 newly developed real-time PCR assays, we screened 496 respiratory specimens from 9 pneumonia-patient populations for APM. This virus was not detected in any specimen, which suggests it is not a common respiratory pathogen.

Investigation of a suspected Legionnaire’s pneumonia outbreak in 1992 led to the isolation of a new microorganism from a water cooling tower in Bradford, England. This pathogen was thought to be a bacterium because it resembled small gram-positive cocci; however, in 2003 it was correctly identified as a virus (*1*). *Acanthamoeba polyphaga mimivirus* (APM), named for its ameba host and bacteria-mimicking characteristics, is a double-stranded DNA virus with the largest viral genome described to date (1.2 Mb) (*2*). *Mimiviridae* is the newest member of the nucleocytoplasmic large DNA virus (NCLDV) group, which also contains *Poxviridae*, *Iridoviridae*, *Asfarviridae,* and *Phycodnaviridae* (*1*). APM encodes specific translation proteins that are more commonly associated with cellular organisms than with viruses (*2*).

Other ameba-associated microorganisms from environmental sources, such as *Legionella pneumophila*, are known to cause outbreaks of acute pneumonia in immunosuppressed and elderly persons, although person-to-person transmission is uncommon. Whether APM is similarly responsible for individual cases or outbreaks of respiratory disease has yet to be conclusively determined. Previous studies have reported serologic evidence of APM infection in 7.1% to 9.7% of patients with community- or nosocomially acquired pneumonia (*3*,*4*). APM DNA was also amplified by a nested PCR assay from a bronchoalveolar lavage specimen of a 60-year-old patient receiving intensive care for hospital-acquired pneumonia (*3*). In this study, we used newly developed real-time PCR assays to screen pneumonia patients from a variety of epidemiologic settings for APM infections.

## The Study

Real-time PCR assays for APM were developed from multiple primers and probes designed for conserved regions of class I NCLDV genes L396 and R596, class III NCLDV gene L65, as well as the R656 gene, from the published APM genome sequence (GenBank accession no. NC_006450) by using Primer Express 3.0 software (Applied Biosystems, Foster City, CA, USA). All probes were labeled at the 5′ end with 6-carboxy-fluorescein and quenched at the 3′ end with Black Hole Quencher-1 (Biosearch Technologies, Novato, CA, USA). Different primer and probe combinations were evaluated, and the 2 PCR assays that gave the best performance were selected for further studies (Table 1). Assays were performed by using the iQSupermix Kit (Bio-Rad, Hercules, CA, USA) in 25-μL reaction volumes. Amplification was performed on an iCycler iQReal-Time Detection System (Bio-Rad) by using the following cycling conditions: 95ºC for 3 min for 1 cycle; 95ºC for 15 s and 55ºC for 1 min for 45 cycles each. Total nucleic acid was extracted from all specimens by using either the NucliSens Automated Extractor (bioMérieux, Boxtel, the Netherlands) or the automated BioRobot MDx (QIAGEN, Valencia, CA, USA) according to the manufacturers’ instructions. Each clinical specimen was also tested for the human ribonuclease P gene to measure nucleic acid integrity as previously described (*5*).

**Table 1:** Primer and probe sequences for *Acanthamoeba polyphaga mimivirus* real-time PCR assays

Target	Primer/probe	Gene region	Sequence (5′→3′)
APM-396	Forward		ACC TGA TCC ACA TCC CAT AAC TAA A
	Reverse	Helicase	GGC CTC ATC AAC AAA TGG TTT C
	Probe		ACT CCA CCA CCT CCT TCT TCC ATA CCT TT
APM-596	Forward		AAC AAT CGT CAT GGG AAT ATA GAA AT
	Reverse	Thiol oxidoreductase	CTT TCC AGT ATC CCT GTT CTT CAA
	Probe		TTC GTC ATA TGC GAG AAA ATG CTA TCC CT

For PCR-positive controls, recombinant plasmids containing APM DNA (kindly provided by Didier Raoult, Unite des Rickettsies, Universite de la Mediterranee, Marseille, France) were constructed. Primer pairs bracketing the L396 and R596 genes were used to amplify 1,560-bp and 879-bp full gene regions, respectively, using 300 nmol/L of forward primers 396 F (5′-TTA ATC ATC TTC CAA AAA ATT TAA TTC-3′) and 596 F (5′-ATG TCG TTA TCA AAA CAA GTA GTT CC-3′), and 300 nmol/L of reverse primers 396 R (5′-ATG GCG AAC AAT ATT AAA ACT AAA A-3′) and 596 R (5′-CTA ATT TTC AAT ATA GTG CGT AGA TTC TA-3′). These PCR products were purified by using the QIAquick Gel Extraction Kit (QIAGEN) and then cloned into a pCR-II TOPO vector by using a TOPO TA Cloning Kit (Invitrogen, Carlsbad, CA, USA). Recombinant plasmids were then isolated by using the QIAprep Spin Miniprep Kit (QIAGEN) and quantified by UV spectroscopy. Standard curves were prepared from serial 10-fold dilutions of the quantified plasmid in nuclease-free water containing 100 μg/mL of herring sperm DNA (Promega, Madison, WI, USA).

**Figure 1:**
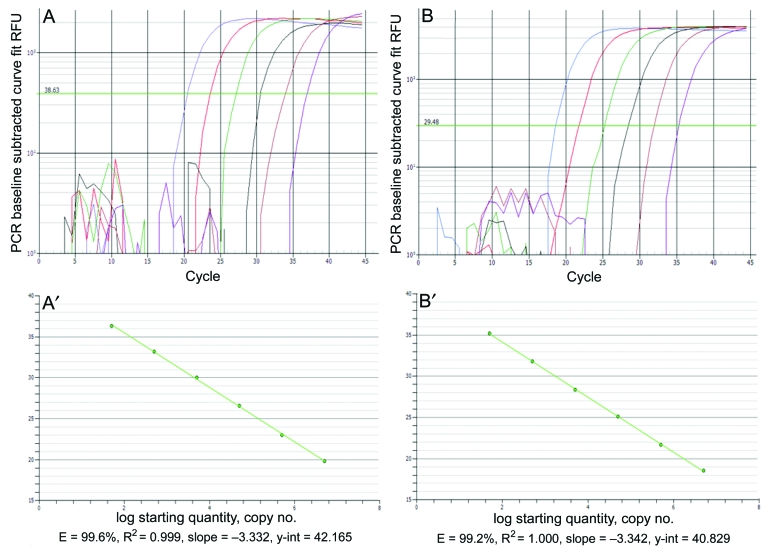
Real-time PCR amplification plots and standard curves for *Acanthamoeba polyphaga mimivirus* (APM)-396 (A, A′) and APM-596 (B, B’). Linear amplification was achieved over 6 logs for both assays over 5 × 10^6^ to 5 × 10^1^ copies of plasmid DNA. RFU, relative fluorescence units.

The L396 and R596 real-time PCR assays could detect as few as 10 copies of plasmid DNA per reaction with amplification efficiencies of 99.6% [slope –3.33 and r^2^ = 0.99] (Figure 1, left panels) and 99.2% [slope –3.34 and r^2^ = 1.00] (Figure 1, right panels), respectively. No amplification was obtained by either assay with pooled total nucleic acid extracts from respiratory samples from healthy humans or from other common DNA respiratory viruses, including adenovirus, human bocavirus, or herpesviruses.

The real-time PCR assays were used to test respiratory specimens from 496 pneumonia cases representing 9 distinct patient populations, which consisted of hospitalized pneumonia patients from population-based pneumonia surveillance studies in Thailand and the United States, transplant recipients with pneumonia, and isolated pneumonia outbreaks in either retirement homes for the elderly or familial clusters (Table 2). Of the 496 specimens tested, no positive results were obtained for APM DNA by either assay.

**Table 2:** Characteristics of 496 pneumonia patients tested for *Acanthamoeba polyphaga mimivirus* DNA*

Setting or population	Sample size	Age group	Sample type	Location	Period	Other causes
Community-acquired pneumonia cases	124	Children<5 y	Nasal swabs	Urban USA	Oct 2000– Sep 2001	None detected
	120	Adults, children	NP swabs	Rural Thailand	Sep 2003– Aug 2004	None detected
Nosocomially acquired pneumonia outbreaks	23	Geriatric	NP/OP swabs	Retirement center, USA	Sep 2003	20% rhinovirus
	24	Geriatric	NP/OP swabs	Retirement center, USA	Jul–Aug 2002	20% rhinovirus
	24	Geriatric	Nasal swabs	Retirement center, USA	May 2004	50% hMPV
Community-acquired pneumonia outbreak	5	Adults, children	BAL, sputum, ET aspirate	Familial cluster, USA	Nov 2004	None detected
Bone marrow transplant recipients	42	Adults	NP aspirate	USA	Jan–Apr 2001	60% other respiratory viruses
	45	Adults	Nasal wash, NP swabs	USA	2003	10% influenza and picornaviruses
Lung transplant recipients	89	Adults	NP swabs	Canada	2002–2003	30% other respiratory viruses

*NP, nasopharyngeal; OP, oropharyngeal; hMPV, human metapneumovirus; BAL, bronchoalveolar lavage; ET, endotracheal.

## Conclusions

We developed a rapid method of screening samples for APM DNA by using 2 sensitive and specific real-time PCR assays designed to target conserved NCLDV class I genes. With only 1 APM sequence published (NC_006450) (*2*), little is known of APM strain variation; therefore, use of assays that target different genes increases the likelihood that genetic variants of APM will not be missed. A suicide-nested PCR method for APM detection has been reported (*3*); however, the quicker turnaround time and lower risk for amplicon contamination makes the real-time PCR method more attractive for screening large numbers of samples.

A seroprevalence study of APM among Canadian patients with community-acquired pneumonia identified APM antibodies in 9.7% of 376 patients compared with 2.3% of 511 healthy controls (*3*). However, seropositivity may reflect exposure to APM antigen rather than active infection, and the potential for nonspecific cross-reactions with the serologic assays used may have inflated the true prevalence of APM infection (*6*). In a separate report, a laboratory-acquired APM infection was linked to acute pneumonia by seroconversion in a technician in Marseille, France, thus providing evidence that this virus can occasionally cause clinical disease (*7*). However, using sensitive real-time PCR assays, we failed to detect APM DNA in 496 respiratory specimens from 9 epidemiologically varied pneumonia patient populations.

If we assume an APM prevalence of 0.2% (1 case in the study sample), the estimated probability of obtaining our results by chance, based on binomial analysis, would be 0.37. Most of the specimens we tested were from the upper respiratory tract, whereas the only reported APM PCR–positive sample was from a lower respiratory bronchoalveolar lavage specimen (*3*). Moreover, the patient populations sampled may not represent those at highest risk for APM infection. Nevertheless, our study supports the findings of an Austrian study that failed to detect APM in 214 nasopharyngeal specimens from hospitalized children with respiratory symptoms (*8*).

Our study did not detect APM in a large collection of specimens from patients with pneumonia, which indicates that this virus is not a common cause of severe acute respiratory disease. Because APM is an ameba-associated pathogen like *Legionella*, exposures to APM are most likely to occur from environmental sources. Further studies of more epidemiologically appropriate populations may be necessary to adequately access the importance of APM as a potential human respiratory pathogen. The real-time PCR assays described here will help facilitate these studies.
